# Linking Thermal Ecology and Agricultural Risk: Generational Potential of *Diceraeus melacanthus* in Southern and Central Brazil

**DOI:** 10.3390/insects16121242

**Published:** 2025-12-09

**Authors:** Luciano Mendes de Oliveira, Rodolfo Bianco, Maurício Ursi Ventura, Ayres de Oliveira Menezes Júnior, Humberto Godoy Androcioli

**Affiliations:** 1 Entomology Laboratory–Instituto de Desenvolvimento Rural do Paraná—IAPAR-EMATER, Highway Celso Garcia Cid, Kilometer 375—Conjunto Ernani Moura Lima II, Londrina 86047-902, PR, Brazil; luciano.agro.oliveira@gmail.com (L.M.d.O.); rbianco@idr.pr.gov.br (R.B.); 2 Agronomy Department, Universidade Estadual de Londrina (UEL), Highway Celso Garcia Cid, Kilometer 380, Londrina 86057-970, PR, Brazil; mventura@uel.br (M.U.V.); ayres@uel.br (A.d.O.M.J.)

**Keywords:** degree-day model, thermal requirements, maize pests, reproductive potential

## Abstract

*Diceraeus melacanthus* (Dallas, 1851) (Hemiptera: Pentatomidae) has become a key pest in Brazilian maize production, particularly during seedling establishment. This study estimated its lower and upper developmental thresholds (Tb and Tsup), thermal constant (K), and degree-day requirements, and used these parameters to model the potential annual generations (PAG) across the Mato Grosso do Sul, Paraná, and São Paulo states. Biological parameters were calculated from controlled laboratory assays, and historical meteorological datasets were combined with regression models and spatial analyses to generate phenology maps of PAG. Results indicated marked regional differences: Mato Grosso do Sul presented the highest potential, averaging eleven generations per year, São Paulo showed intermediate values with nine generations, and Paraná exhibited the lowest, with approximately seven generations annually. Latitude exerted the strongest influence on PAG, while altitude contributed the least. These findings are consistent with the known adaptability of *D. melacanthus* to warmer climates and highlight its capacity to persist in no-tillage soybean–maize systems and areas with volunteer plants. The results provide a predictive framework for assessing population risk and may support decision-making in integrated pest management. Further studies on host range, phenology, and distribution are required to anticipate future expansions across South America.

## Simple Summary

The green-belly stink bug (*Diceraeus melacanthus*), as it is known in Brazil, is currently one of the most important maize pests in Brazil, especially during the early stages of the crop. This study evaluated if geographic characteristics influence *D. melacanthus* development, and estimated the potential annual generations in the states of Mato Grosso do Sul, Paraná, and São Paulo. Laboratory experiments determined the minimum and maximum developmental thresholds and the degree-day requirements for the species, which were then combined with historical climate data and topographic to generate phenology maps showing the potential number of generations across different regions. Results revealed that each state’s topography and climate influenced *D. melacanthus* potential annual generations. Latitude and altitude strongly influenced these patterns, with warmer and lower-altitude areas showing greater reproductive potential. These findings help explain the current distribution of the pest and highlight its adaptability to different environments. The results also provide useful information for farmers and technicians to better anticipate risks and improve integrated pest management strategies, particularly through seed treatment and careful monitoring of field populations.

## 1. Introduction

Understanding the ecology and biology of insect pests is fundamental for developing effective management strategies [1,2,3]. Abiotic factors such as temperature, humidity, and photoperiod exert direct effects on insect performance, viability, development, and survival [4]. Among the main pest species, the green-belly stink bug *Diceraeus melacanthus* (Dallas, 1851) (Heteroptera: Pentatomidae) is an hemimetabolous (five nymphal instars) and polyphagous insect capable of feeding on several crops, starting from early nymphal instars, particularly soybean [*Glycine max* (L.) Merr.], maize (*Zea mays* L.), and wheat (*Triticum aestivum* L.) [1,5,6,7]. These crops are cultivated throughout the year in the states of Mato Grosso do Sul, Paraná, and São Paulo, with wheat production being especially relevant in Paraná state. As a result, *D. melacanthus* has continuous feeding opportunities across these agricultural regions [8].

Climatic conditions in these states are diverse. Paraná is located in a transition zone between a humid subtropical climate with hot summers (Cfa) and a temperate humid climate with mild summers (Cfb). Mato Grosso do Sul is predominantly characterized by a tropical wet climate with rainy summers and dry winters (Aw). São Paulo is mainly classified as humid subtropical with dry winters and rainy summers (Cwa). These acronyms are in accordance with the Köppen–Geiger climatic classifications [9]. These climatic variations support the cultivation of crops with distinct thermal and hydric requirements [8,10]. At the same time, they influence insect distribution by restricting the availability of host plants or imposing temperature conditions that may prevent insects from completing their developmental cycle.

Successful insect development requires favorable combinations of nutrient availability, moisture, and temperature [11]. Photoperiod and temperature are closely linked to insect metabolism, and each species exhibits specific thresholds that determine the duration of its life cycle [4]. For *D. melacanthus*, the average growth cycle is 27 days at 25 °C, becoming shorter at higher temperatures (31 °C) and longer at lower ones (16 °C); this is directly influenced by the upper and lower developmental base temperatures [12,13]. The reproductive potential of *D. melacanthus* is relatively unknown. Such knowledge is essential to explain population outbreaks in some regions and the absence of this species in others.

The potential number of insect generations can be represented using climatic suitability maps, which identify areas with higher or lower generational frequency [14,15,16,17,18]. These maps are constructed from historical temperature records and are spatially explicit, considering latitude, longitude, and altitude. The construction and availability of these climatic suitability maps enhance current and future integrated pest management (IPM) programs. Understanding pest ecology and thermal biology is key for the proper performance of IPM strategies, given that insect adaptability and survival influence potential plant damage and production loss [15,16]. Maps that represent insect phenology may also be used to guide pest management decision-making [19].

The objective of this study was to determine the lower (Tb) and upper (Tup) developmental base temperatures, the thermal constant (K), and degree-days for *D. melacanthus*, then to verify if geographic characteristics influence the specimen’s development, and finally, to estimate the potential annual generations in the states of Mato Grosso do Sul, Paraná, and São Paulo, represented with phenology maps.

## 2. Materials and Methods

### 2.1. Study Environment

The data used in this study were obtained from previously published research, which investigated the biology of *Diceraeus melacanthus* through two experimental trials (2017 and 2019) [13]. In that work, the green-belly stink bug was reared under five constant temperatures (16, 21, 26, 31, and 36 °C ± 1 °C) with a 14:10 h (light/dark) photoperiod in climate-controlled chambers and was fed with peanut seeds (*Arachis hypogaea*), soybean grains, and bean pods (*Phaseolus vulgaris*).

### 2.2. Evaluation Concept

Ecdysis, instar change, and total development time for *D. melacanthus* were tabulated (Table 1).

Based on the total developmental period of *D. melacanthus*, the following biological parameters were calculated: lower developmental threshold (Tb), upper developmental threshold (Tup), and thermal constant (K). The biofix was set at egg hatching. Subsequently, values of degree-days (DD), accumulated degree-days (ADD), and the potential annual generations (PAG) were estimated. These data were later used to construct phenology maps showing the potential generations of *D. melacanthus* in the states of Mato Grosso do Sul, Paraná, and São Paulo.

Meteorological data for the state of Mato Grosso do Sul were obtained from the online database of the National Institute of Meteorology (INMET), comprising historical records (2008–2023) from 23 municipalities: Água Clara, Amambai, Aquidauana, Bataguassu, Campo Grande, Cassilândia, Corumbá, Costa Rica, Coxim, Dourados, Itaquirai, Ivinhema, Jardim, Juti, Maracaju, Miranda, Ponta Porã, Porto Murtinho, Rio Brilhante, São Gabriel do Oeste, Sete Quedas, Sonora, and Três Lagoas.

Meteorological data for the state of Paraná were provided by the Meteorology Department of the Instituto de Desenvolvimento Rural do Paraná IAPAR-EMATER (IDR-Paraná). The dataset covered a 39-year period (1976–2015) across 28 municipalities: Antonina, Bandeirantes, Bela Vista do Paraíso, Cambará, Cândido de Abreu, Cascavel, Cerro Azul, Clevelândia, Fernandes Pinheiro, Francisco Beltrão, Guarapuava, Guaraqueçaba, Ibiporã, Joaquim Távora, Lapa, Londrina, Morretes, Nova Cantú, Palmas, Palotina, Paranavaí, Pato Branco, Pinhais, Planalto, Ponta Grossa, São Miguel do Iguaçu, Telêmaco Borba, and Umuarama. The 2015 cut-off was due to missing records and municipalities in later years.

Meteorological data for the state of São Paulo were also obtained from the INMET database, comprising records from 2003 to 2023 for 31 municipalities: Ariranha, Avaré, Barra Bonita, Barra do Turvo, Barretos, Barueri, Bauru, Campos do Jordão, Casa Branca, Franca, Ibitinga, Iguape, Itapeva, Itapira, Ituverava, Jales, José Bonifácio, Lins, São Paulo, Ourinhos, Piracicaba, Pradópolis, Presidente Prudente, Rancharia, São Carlos, São Luís do Paraitinga, São Miguel Arcanjo, Sorocaba, Taubaté, Valparaíso, and Votuporanga.

The latitude, longitude, and altitude of each municipality corresponded to the location of its respective meteorological station.

### 2.3. Data Analysis

Given the limited available data on *D. melacanthus* development in different temperatures, the lower base temperature (Tb) was calculated using a linear regression where the abscissa represented the rearing temperatures and the ordinates represented the development rate (DR).

DR = 1 ÷ each development stage duration (days),(1)

The Tb is found where the linear regression crosses the abscissa, in other words, where the DR is null.

The thermal constant (K) was found through the linear regression’s angular coefficient (b) multiplicative inverse.

K = b^−1^ (2)

The upper base temperature was obtained through the sum of the Tb with the square root of the thermal constant.

Tup = Tb + √K(3)

The optimal temperature range for development was determined with the following formulas, resulting in the upper and lower limits (UL and LL):

UL (°C) = Tb + √(Tb^2^ + 4 × K) ÷ 2, (4)

LL (°C) = Tb + K^1/4^ (5)

The required development days for the upper limit are equal to its value, but the units are in days. To calculate the required development days for the inferior limit, the following formula was used:

LL required development days = (K^3^)^1/4^ (6)

The degree-days were calculated following the method as shown by De Melo, Tenente, and de Olivera [20]:

(7) $$if:\;T_{min}>T_{b}\;and\;T_{max}<T_{up},\;Then\;DD\;=\frac{T_{max}+T_{min}}{2}-T_{b}$$

(8) $$if:\;T_{min}<T_{b}\;and\;T_{max}<T_{up},\;Then\;DD=\frac{{\left(T_{max}+T_{b}\right)}^{2}}{2\left(T_{max}-T_{min}\right)}$$

(9) $$\begin{array}{l} if:\;T_{min}>T_{b}\;and\;T_{max}>T_{up},\;Then\;DD\\ =\frac{2\left(T_{max}-T_{min}\right)\times \left(T_{min}-T_{b}\right)+{\left(T_{max}-T_{min}\right)}^{2}-{\;\left(T_{max}-T_{up}\right)}^{2}\;}{2\left(T_{max}-T_{min}\right)} \end{array}$$

(10) $$ADD=\sum_{i=1}^{n}{DD}_{i}$$

where

*DD* = Degree-days

*ADD* = Accumulated degree-days

*T_max_* = Maximum temperature recorded during the day

*T_min_* = Minimal temperature recorded during the day

*T_b_* = Lower base organism development temperature

*T_up_* = Upper base organism development temperature

*n* = Number of accumulated days

*i* = Index variable of number of days used in degree-day calculation

The degree-day (DD) calculation was accomplished using the averaged biological parameters (Table 2). The DD for each state’s municipality was calculated, and their sum represented the municipality’s accumulated degree-days (ADD). This value was then divided by the total years of historical meteorological data, resulting in the average municipality accumulated degree-days (AMADD). Finally, these means were divided by the thermal constant (K), resulting in the municipality’s potential annual generations (PAG); these results are represented in tables for each state.

The PAG results were then extrapolated to cover each state utilizing a linear multiple regression, considering the variations in latitude (decimal degrees), longitude (decimal degrees), and altitude (meters):

a + (b × Latitude) + (c × Longitude) + (d × Altitude)(11)

where a = equation constant; b = latitude factor variable; c = longitude factor variable; and d = altitude factor variable.

Three linear multiple regressions were created, one for each state, and each of these regressions was calculated utilizing the latitude, longitude, and altitude of the previously cited municipalities.

### 2.4. Software Used

The biological temperature standards, degree-day macros, and multiple linear regression were all calculated utilizing the Microsoft Office Excel (Microsoft 365) software. The macros were used to automate degree-day calculation by using recorded daily temperatures, present in several datasheets from varying dates, and automating data cross-referencing. The linear multiple regressions were calculated utilizing the Office Excel function of INDEX combined with LINEST. The *D. melacanthus* phenology maps were later produced utilizing the free-access QGIS (v3.28.12) software [21]. Potential annual generations (PAG) and average municipal accumulated degree-days (AMADD), derived from 25 randomly selected municipalities, for the Mato Grosso do Sul, Paraná, and São Paulo states, were analyzed for normality, homoscedasticity, and ANOVA mean comparisons, following Scott–Knott (α < 0.01), using the free-access Sisvar software (version 5.8) [22].

### 2.5. QGIS Methodology and Operation

First, the Coordinate Reference System (CRS) was standardized to the World Geodetic System (WGS 1984), authority identifier EPSG:4326. Elevation data for the three states were obtained from NASA’s global topographic program, the Shuttle Radar Topography Mission (SRTM) [23]. Elevations were represented as raster layers in Float32 (GeoTIFF) format for each state using the SRTM-Downloader plugin, developed by Dr. Horst Düster. Each elevation raster layer was subsequently clipped to the boundary of the respective state using the “clip raster by mask layer” tool. The mask layers were territorial grids of federal units (released in 2022) for the states of Mato Grosso do Sul, Paraná, and São Paulo, provided by the Brazilian Institute of Geography and Statistics (IBGE).

Latitude and longitude data for each state were obtained by importing a Comma-Separated Values (CSV) file into QGIS through the Data Source Manager. The file was initially introduced as delimited text with geometry defined by latitude and longitude values in the respective columns. Latitude and longitude values were stored in the attribute table in decimal degrees. This file was then exported as a GeoPackage to facilitate further manipulation. Next, rasterization of these data was performed for each GeoPackage file to generate individual raster layers of latitude and longitude for each state. This was accomplished using the SAGA GIS Provider plugin, developed by Víctor Olaya, applying its polynomial regression function configured to produce a simple planar surface (GeoTIFF) with 1000 × 1000 pixels.

Finally, multiple linear regressions generated from biological and meteorological data for each state were applied using the QGIS raster calculator, in which latitude, longitude, and altitude were represented by their respective raster layers (Float32). This process resulted in a Float32 file containing the extrapolated regression outputs across the entire state territory. These files were subsequently symbolized to display fluctuations in the potential number of *D. melacanthus* annual generations in each state. Visualization was performed using single-band false-color rendering with discrete interpolation, an inverted spectral color gradient, and five classes defined. The maps for each state were ultimately plotted both individually and jointly, with the scale of potential annual generations of *D. melacanthus* standardized across all outputs.

## 3. Results

**Figure 1:**
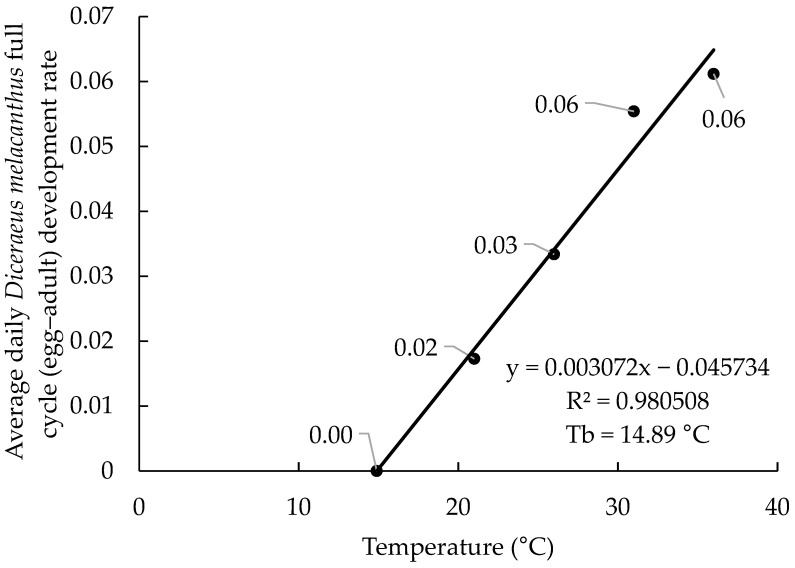
Linear regression used to determine the lower base temperature (Tb) of *Diceraeus melacanthus* using the 2017 dataset rearing temperature and development rate (DR). Londrina, Paraná state, IDR-Paraná, 2025.

**Figure 2:**
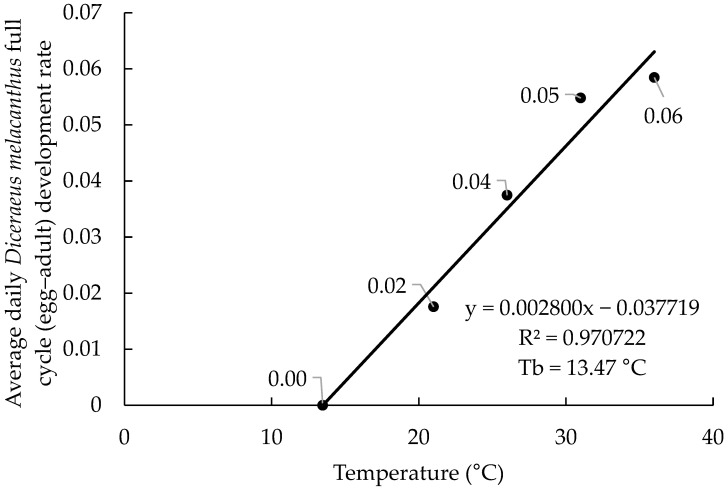
Linear regression used to determine the lower base temperature (Tb) of *Diceraeus melacanthus* using the 2019 dataset rearing temperature and development rate (DR). Londrina, Paraná state, IDR-Paraná, 2025.

The linear regressions based on the 2017 and 2019 datasets showed highly consistent results, with coefficients of determination (R^2^) of 0.98 and 0.97, respectively (Figure 1 and Figure 2). The estimated equations were (−0.045734 + 0.003072x) for 2017 and (−0.037719 + 0.002800x) for 2019. The lower base organism development temperature (Tb) was calculated at 14.89 °C (2017) and 13.47 °C (2019), indicating similar biological requirements. In both years, the highest developmental rates were observed at 31 and 36 °C, ranging from 0.055 to 0.061 day^−1^. The marginal differences in developmental rate between the 31 and 36 °C temperatures (0.006 day^−1^ in 2017 and 0.003 day^−1^ in 2019) may suggest that these are neighboring the upper base organism development temperature (Tup).

The biological parameters estimated from the 2017 and 2019 datasets were highly consistent, with only minor variations between years (Table 2). The lower base temperatures (Tb) were 14.89 °C in 2017 and 13.47 °C in 2019, while the Tup was nearly identical, at 32.9 °C and 32.4 °C, respectively. The thermal constant (K) showed the largest difference, with 325.5 degree-days in 2017 and 357.1 degree-days in 2019, indicating a longer developmental duration in 2019. The upper limit of the optimal temperature range (UL) was stable across years (27 °C, 27 days), whereas the lower limit (LL) showed greater variation, with 19.1 °C and 77 days in 2017 and 17.8 °C and 82 days in 2019. The UL and LL serve to represent the best development range for the specimen; they do not represent development thresholds. The thresholds are represented by the Tb and Tup values. Together, these values corroborate the regression analyses, reinforcing that 31 and 36 °C are near the Tup.

In the state of Mato Grosso do Sul, the potential annual generations (PAG) of *D. melacanthus* was the highest among the three states, averaging approximately 11 generations per year, with an average municipal accumulated degree-days (AMADD) value of about 3751 (Table 3). The municipality of Corumbá presented the highest PAG, with a potential of thirteen generations annually, whereas the lowest values, nine generations per year, were recorded in Amambai, Ponta Porã, and Sete Quedas. It is worth noting that the municipalities of Aquidauana, Anastácio, Corumbá, Dois Irmãos do Buriti, Ladário, Miranda, and Porto Murtinho have little agronomic relevance in the state.

The PAG of *D. melacanthus* for Mato Grosso do Sul was estimated using the following values in Equation (11): a = 25.6611; b = 0.4035; c = 0.0836; d = −0.0043, with a determination coefficient (R^2^) of 0.93. The intercept (a) was relatively high, approximately 26, with the greatest contribution from latitude (b ≈ 0.4) and the lowest from altitude (c ≈ 0.08). Altitude tends to contribute less than the other variables due to the wide variation within the state, ranging from 100 m in the Pantanal lowlands to 1160 m in Maciço do Urucum. No significant difference was found between the PAG calculated using degree-days and the linear multiple regression estimated PAG.

In Paraná state, *D. melacanthus* exhibited the lowest mean PAG among the three states, with approximately seven generations per year and an AMADD of about 2482 (Table 4). The municipality of Paranavaí showed the highest potential, with ten generations annually, whereas the lowest values, four generations per year, were observed in Lapa, Palmas, and Pinhais. It is important to note that the municipalities along the coastal plain of Paraná—Antonina, Guaraqueçaba, Guaratuba, Matinhos, Morretes, Paranaguá, and Pontal do Paraná—are not agriculturally relevant for maize production in the state.

The *D. melacanthus* PAG in Paraná was obtained using Equation (11) with the following parameters: a = 13.5979; b = 0.8341; c = −0.3306; d = −0.0045 (R^2^ = 0.93). The intercept (a) was the lowest among the three states, approximately 14. Once again, latitude (b) had the strongest positive contribution (≈0.83), while longitude (c) contributed negatively (≈−0.33). As expected, altitude had the smallest effect, given its wide variation in the state, from sea level in the coastal region to 1877 m at Pico Paraná in Campina Grande do Sul. No significant difference was found between the PAG calculated using degree-days and the linear multiple regression-estimated PAG.

In São Paulo, *D. melacanthus* presented an average of approximately nine generations per year, with an AMADD of 2995 (Table 5). The municipalities of Jales, José Bonifácio, Lins, Presidente Prudente, Valparaíso, and Votuporanga showed the highest PAG values, with up to eleven generations annually, whereas the lowest value, three generations per year, was recorded in Campos do Jordão. It is important to highlight that the mesoregions of the southern seaside and the São Paulo metropolitan region have no significant agricultural relevance in the state.

The PAG of *D. melacanthus* in São Paulo was estimated using Equation (11) with the following parameters: a = 22.4430; b = 0.8452; c = −0.1589; d = −0.0040 (R^2^ = 0.90). The intercept (a) represented an intermediate value among the three states, approximately 22. Once again, latitude (b ≈ 0.84) showed the strongest positive effect, whereas longitude (c ≈ −0.15) contributed negatively. Altitude had the smallest effect, as expected, due to its wide range in the state, from sea level along the coast to 2798 m in the Serra da Mantiqueira region. No significant difference was found between the PAG calculated using degree-days and the linear multiple regression-estimated PAG.

The comparative analysis among the three states revealed clear differences in *D. melacanthus* PAG (Table 6 and Table 7). The data was considered normally distributed in accordance with the Kolmogorov–Smirnov test (α < 0.01) and adequate homoscedasticity (Table 6). The PAG ANOVA analysis presented significant (F α < 0.01 = 4.91) variations between each state (F = 34.6). The AMADD ANOVA analysis also found significant (F α < 0.01 = 4.91) variations between each state (F = 34.6). Mato Grosso do Sul exhibited the highest values, with a mean of approximately 11 generations and an AMADD of about 3751, both statistically significant (α < 0.01). São Paulo showed intermediate values, averaging 9 generations annually and an AMADD of 2995, while Paraná presented the lowest estimates, with a mean of approximately 7.25 generations and an AMADD of 2473, also significant at α < 0.01. In all models, latitude exerted the strongest positive effect, while altitude contributed the least, reflecting the wide altitudinal ranges within each state. These findings indicate that climatic and topographic gradients strongly influence the potential population growth of *D. melacanthus*, with the warmer and lower-altitude regions of Mato Grosso do Sul and western São Paulo offering the most favorable conditions for higher generational turnover, whereas Paraná presents more restrictive environments for population expansion

**Figure 3:**
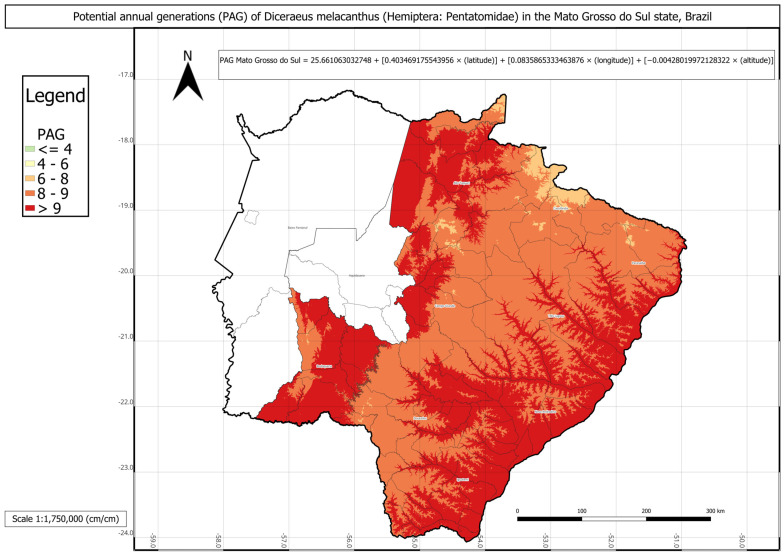
Phenology map representing *Diceraeus melacanthus* potential annual generations (PAG) in the Mato Grosso do Sul state, Brazil, and the multiple linear regression used to construct the map. The municipalities of Aquidauana, Anastácio, Corumbá, Dois Irmãos do Buriti, Ladário, Miranda, and Porto Murtinho have little agronomic relevance in the state. Londrina, Paraná state, IDR-Paraná, 2025.

**Figure 4:**
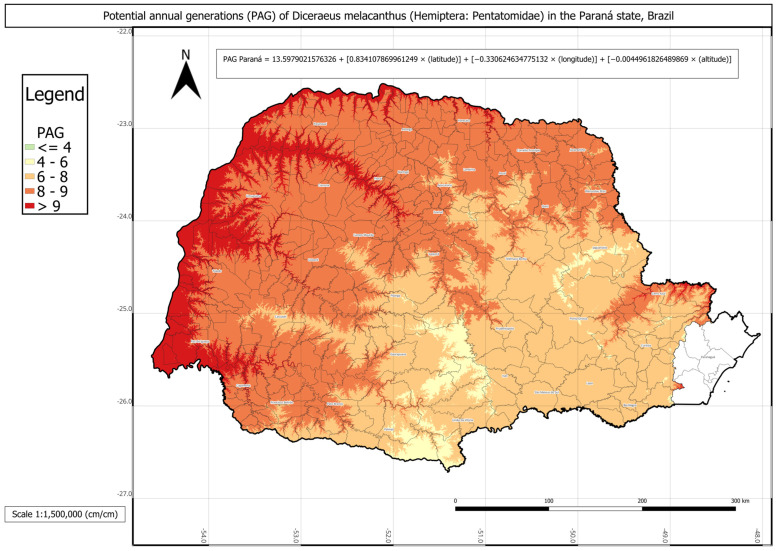
Phenology map representing *Diceraeus melacanthus* potential annual generations (PAG) in the Paraná state, Brazil, and the multiple linear regression used to construct the map. The municipalities Antonina, Guaraqueçaba, Guaratuba, Matinhos, Morretes, Paranaguá, and Pontal do Paraná are not agriculturally relevant for maize production in the state. Londrina, Paraná state, IDR-Paraná, 2025.

**Figure 5:**
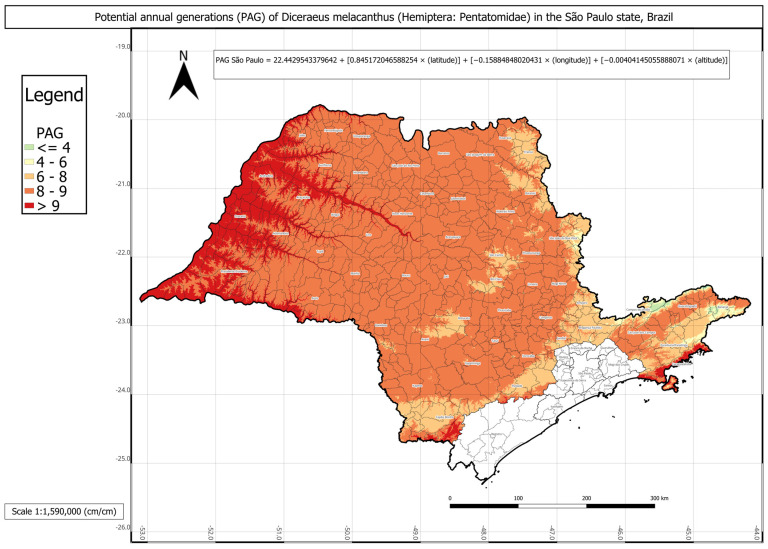
Phenology map representing *Diceraeus melacanthus* potential annual generations (PAG) in the São Paulo state, Brazil, and the multiple linear regression used to construct the map. The mesoregions of the southern seaside and the São Paulo metropolitan region have no significant agricultural relevance in the state. Londrina, Paraná state, IDR-Paraná, 2025.

**Figure 6:**
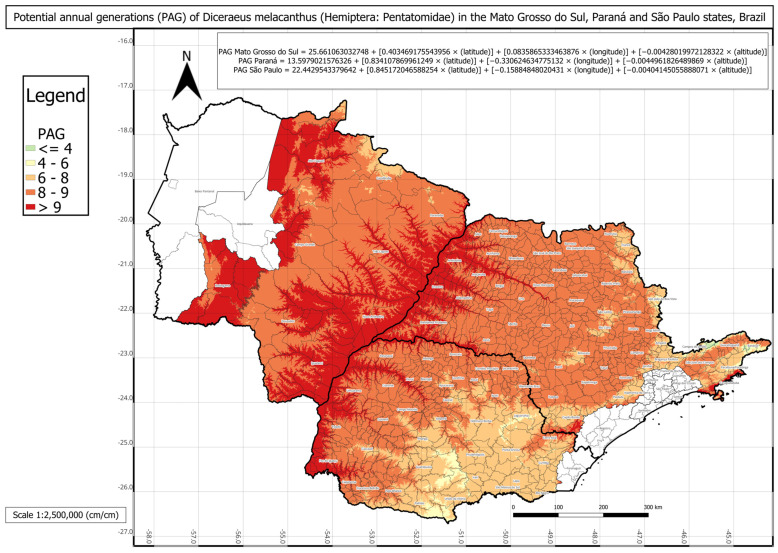
Phenology map representing *Diceraeus melacanthus* potential annual generations (PAG) in the Mato Grosso do Sul, Paraná, and São Paulo states, Brazil, and the multiple linear regressions used to construct the map. The uncolored regions have no significant agricultural relevance. Londrina, Paraná state, IDR-Paraná, 2025.

The statistical comparison between PAG values obtained directly from historical climate records and those estimated using the respective multivariate regressions for each state showed no significant differences. The regression-based PAG models were represented in three individual phenology maps for each state and one combined map for all three states (Figure 3, Figure 4, Figure 5 and Figure 6).

The phenology map analysis revealed distinct spatial patterns of *D. melacanthus* PAG across the three states. Mato Grosso do Sul consistently exhibited the highest PAG values, with most municipalities sustaining more than eight generations per year and several exceeding nine, particularly along the borders with Paraná, São Paulo, and Paraguay (Figure 3). In contrast, Paraná showed the lowest overall PAG values, averaging between four and eight generations in the central, southern, and metropolitan regions, while values above nine were restricted to municipalities bordering Paraguay, Argentina, and northern São Paulo (Figure 4). São Paulo state presented intermediate conditions, with a PAG above eight in most mesoregions, except in the Vale do Paraíba Paulista, where values fell below four, especially in Bananal and Campos do Jordão (Figure 5). The highest PAG values in São Paulo were concentrated in the western regions bordering Mato Grosso do Sul and Paraná. These results demonstrate the strong influence of latitude, altitude, and regional climatic gradients on the distribution of *D. melacanthus* generational potential, with warmer, lower-altitude regions favoring higher PAG and cooler, elevated areas acting as natural constraints (Figure 6).

## 4. Discussion

Degree-days is a useful estimation, calculated through the combination of time and temperature; it ultimately represents the organism development rate during specific phenological stages. The degree-days calculated for the *Diceraeus melacanthus* potential annual generations (PAG) was accomplished following the method discussed by De Melo, Tenente, and de Olivera [20]. This method was chosen because of its high precision, since it considers daily temperature fluctuations and specimen-specific lower and upper temperature thresholds [20]. It is important to highlight that all these calculations are based on data from insects reared in constant temperatures; although this is not common in nature, it is common in controlled insect rearing conditions, which offer the best conditions for proper development, greater viability, and healthy reproduction [13]. Food availability, survivability, and biological control were not evaluated during this study.

The results of this study demonstrate that the potential annual generations of *D. melacanthus* is strongly influenced by climatic gradients, particularly temperature and altitude, which is consistent with patterns reported for other Pentatomidae species. Previous research has shown that thermal requirements and degree-day models are effective tools for predicting population dynamics of stink bugs in different agroecosystems [24,25,26]. The higher PAG observed in Mato Grosso do Sul and western São Paulo reflects the predominance of warmer climates and lower altitudes, which accelerate developmental rates and shorten generation times. In contrast, the lower PAG in Paraná, especially in cooler and elevated regions, indicates natural climatic restrictions to population growth. These findings highlight the importance of regional climatic conditions in shaping the distribution and potential risk of *D. melacanthus* infestations across major agricultural areas. It is important to highlight that the data and maps produced in this study were generated considering *D. melacanthus* biological data, and regional meteorological standards, insect survivability, and behavior were not considered. Also, the data and maps represent the PAG values assuming optimal development and survivability conditions. Thus, further research should be conducted examining true *D. melacanthus* migration, survivability, and establishment throughout areas with agricultural importance.

*Diceraeus melacanthus* was first described in Venezuela [27,28]. Initially regarded as a secondary pest of soybean in Brazil, it later became recognized as a key pest in the early developmental stages of maize [5,29]. The first record of damage caused by this species to maize seedlings in Brazil occurred in 1993 in Rio Brilhante, Mato Grosso do Sul [30]. Currently, the insect is widely distributed across Brazil, with higher incidence in states of major agricultural importance, including Goiás, Mato Grosso, Mato Grosso do Sul, Minas Gerais, Paraná, and São Paulo. The expansion and persistence of its populations have been favored by factors such as the adoption of no-tillage systems in soybean–maize rotations, which enhance survival conditions, and the use of transgenic maize cultivars, either Bt or glyphosate-tolerant, which reduced insecticide applications and hindered control of volunteer maize plants [1,31].

Additional factors contributing to the persistence of *D. melacanthus* populations include seed and ear losses during harvest and transport, as well as irregular germination, which promote volunteer plants in fields, roadsides, and adjacent areas. These hosts ensure population continuity during both crop and off-season periods. In this context, maize seed treatment has become fundamental to reducing damage in the early stages of germination. Conversely, pre-emergence insecticide applications have shown low efficacy, and post-emergence interventions are recommended only when population levels exceed 0.8 insects per linear meter [32].

It is important to note that another species of the same genus, *Diceraeus furcatus* (F., 1775) (Hemiptera: Pentatomidae), also has agricultural significance. This species occurs more frequently in southern Brazil and Argentina, possibly due to differences in thermal requirements [33]. Comparative studies on both species are therefore essential to better understand the distribution and reproductive potential of the genus *Diceraeus* in South America.

From a comparative perspective, the brown stink bug *Euschistus heros*, a major soybean pest, develops optimally at temperatures between 22 and 28 °C, whereas *D. melacanthus* exhibits greater adaptability to warmer regions [13,18,24]. Both species share a lower developmental threshold close to 14 °C, but *D. melacanthus* maintains higher developmental rates above 28 °C, unlike *E. heros* (Table 2). The brown marmorated stink bug *Halyomorpha halys* (Stål, 1855) (Hemiptera: Pentatomidae), in turn, requires 537.6 degree-days to complete its life cycle, a value significantly higher than that of *D. melacanthus*, reflecting interspecific differences in thermal requirements [34]. The upper base organism development temperature (Tup) represents the temperature threshold at which the specimen still develops healthy and may reproduce; temperatures above this threshold may result in maldevelopment and infertile adults [13].

The findings of the present study may serve as a basis for risk assessment of maize damage, particularly in high-reproductive-potential regions, as border areas between the three states showed the greatest PAG values (Figure 6). Additional information on the phenology of *D. melacanthus* may support the identification of critical cultivation periods and guide decision-making, similar to initiatives such as the National Phenology Network [19]. According to Tonnang et al. (2015), insect phenological mapping also requires knowledge of diet and alternative host plants [35]. For *D. melacanthus*, alternative hosts include soybean, wheat, and certain weeds such as *Commelina benghalensis* and *Crotalaria lanceolata* [6,36].

Finally, it is noteworthy that the three states analyzed—Mato Grosso do Sul (central-west), São Paulo (southeast), and Paraná (south)—not only represent major agricultural regions but also encompass nationally relevant biomes: Cerrado, Floresta atlântica, and Pantanal, which account for approximately 23%, 13%, and 2% of Brazil’s territory, respectively. These states also represent four distinct Köppen climate types: Mato Grosso do Sul is predominantly tropical wet with rainy summers and dry winters (Aw); Paraná lies in a transition between humid subtropical with hot summers (Cfa) and temperate humid with mild summers (Cfb); and São Paulo is mainly humid subtropical with dry winters and rainy summers (Cwa) [9]. The ability of *D. melacanthus* to adapt across such diverse climatic and ecological zones underscores the need for further studies to assess its potential expansion not only to other Brazilian regions but also to neighboring South American countries such as Argentina, Bolivia, Colombia, Paraguay, and Venezuela.

This study determined the lower and upper developmental thresholds, thermal constant, and degree-day requirements of *Diceraeus melacanthus* and used these parameters to estimate the potential annual generations across the Mato Grosso do Sul, Paraná, and São Paulo states in Brazil. The results demonstrated clear regional differences, with higher generational potential in the warmer, low-altitude areas of Mato Grosso do Sul and western São Paulo, and lower values in cooler, elevated regions of Paraná. The adaptability of *D. melacanthus* to diverse climatic conditions highlights its importance as a key pest in maize and reinforces the need for integrated management strategies. These findings provide a valuable basis for predicting population risk and guiding control decisions, while also emphasizing the necessity of further studies on insect pest distribution, host range, and phenology across agriculturally important territories.

## Figures and Tables

**Table 1:** Development period in days of *Diceraeus melacanthus* hatching, molting and maturing. Biological data obtained from insects reared in constant temperatures. Londrina, Paraná, IDR-Paraná, 2025.

Rearing Year	2017				2019			
Temperature (°C)/Life stages (days)	21	26	31	36	21	26	31	36
Hatching	8	5	3	3	8	5	4	3
1st to 2nd instar	6	3	2	2	5	3	1	1
2nd to 3rd instar	10	6	4	3	11	5	4	4
3rd to 4th instar	9	5	2	2	8	4	3	2
4th to 5th instar	11	5	3	2	12	4	3	3
5th instar to Adult	14	7	4	4	13	6	4	3
Complete cycle (egg–adult)	58	30	18	16	57	27	18	17

Source: de Oliveira et al. [13].

**Table 2:** *Diceraeus melacanthus* biological parameters demonstrating thermal constant (K), lower base temperature (Tb), upper base temperature (Tup), upper development limit (UL) in degrees and days, and lower development limit (LL) in degrees and days; data obtained from the 2017 and 2019 rearing datasets. Londrina, Paraná state, IDR-Paraná, 2025.

Year	K (Degree-Days)	Tb (°C)	Tup (°C)	UL (°C)	UL (Days)	LL (°C)	LL (Days)
2017	325.5	14.9	32.9	27	27	19.1	76.6
2019	357.1	13.5	32.4	26.8	26.8	17.8	82.2
**Average**	341.2	14.2	32.65	26.9	26.9	18.4	79.4

**Table 3:** *Diceraeus melacanthus* potential annual generations (PAG) and average municipal accumulated degree-days (AMADD) for 25 municipalities in the Mato Grosso do Sul state, Brazil; temperature data collected from 15 years of weather registry. Londrina, Paraná state, IDR-Paraná, 2025.

Mato Grosso Do Sul Municipalities	PAG	AMADD	Latitude	Longitude	Altitude
Água Clara	12	4015	−20.44	−52.88	338
Amambai	9	3242	−23.00	−55.33	431
Aquidauana	12	4218	−20.48	−55.78	155
Bataguassu	11	3770	−21.75	−52.47	387
Bela Vista	11	3756	−22.10	−56.54	208
Cassilândia	11	3782	−19.12	−51.72	516
Chapadão do Sul	10	3477	−18.80	−52.60	818
Corumbá	13	4400	−19.00	−57.64	126
Costa Rica	11	3682	−18.82	−53.27	730
Coxim	12	4160	−18.51	−54.74	252
Dourados	10	3391	−22.19	−54.91	469
Itaquiraí	10	3521	−23.45	−54.18	336
Ivinhema	11	3713	−22.31	−53.83	373.8
Jardim	12	3957	−21.48	−56.14	249
Juti	10	3570	−22.86	−54.61	379
Miranda	12	3975	−20.38	−56.42	140
Paranaíba	11	3902	−19.70	−51.18	424
Ponta Porã	9	3049	−22.55	−55.72	675
Porto Murtinho	12	4000	−21.71	−57.89	85
Rio Brilhante	11	3630	−21.77	−54.53	329
São Gabriel do Oeste	10	3570	−19.42	−54.55	647
Sete Quedas	9	3224	−23.97	−55.02	402
Sidrolândia	11	3762	−20.98	−54.97	464
Sonora	12	4007	−17.90	−54.45	486
Três Lagoas	12	3998	−20.79	−51.71	313
Average	11	3751	-	-	-

**Table 4:** *Diceraeus melacanthus* potential annual generations (PAG) and average municipal accumulated degree-days (AMADD) for 28 municipalities in the Paraná state, Brazil; temperature data collected from 39 years of weather registry. Londrina, Paraná state, IDR-Paraná, 2025.

Paraná Municipalities	PAG	AMADD	Latitude	Longitude	Altitude
Antonina	8	2776	−25.22	−48.80	60
Bandeirantes	9	3147	−23.10	−50.35	440
Bela Vista do Paraíso	9	2924	−22.95	−51.20	600
Cambará	9	3121	−23.00	−50.03	450
Cândido de Abreu	6	1879	−24.63	−51.25	645
Cascavel	7	2448	−24.88	−53.55	660
Cerro Azul	8	2803	−24.82	−49.25	360
Clevelândia	5	1651	−26.24	−52.35	930
Fernandes Pinheiro	5	1783	−25.45	−50.58	893
Francisco Beltrão	7	2252	−26.08	−53.07	650
Guarapuava	5	1633	−25.38	−51.50	1026
Guaraqueçaba	8	2798	−25.27	−48.53	40
Ibiporã	9	3095	−23.27	−51.02	484
Joaquim Távora	9	2934	−23.50	−49.95	512
Lapa	4	1214	−25.78	−49.77	910
Londrina	8	2786	−23.37	−51.17	585
Morretes	8	2826	−25.50	−48.82	10
Nova Cantu	8	2884	−24.67	−52.57	540
Palmas	4	1392	−25.24	−51.98	1100
Palotina	9	3061	−23.52	−53.92	310
Paranavaí	10	3286	−24.39	−52.43	480
Pato Branco	6	2116	−24.63	−52.68	700
Pinhais	4	1512	−23.00	−49.13	930
Planalto	8	2888	−24.89	−53.78	400
Ponta Grossa	5	1841	−24.83	−50.02	880
São Miguel do Iguaçu	9	3086	−25.45	−54.37	260
Telêmaco Borba	6	2167	−25.55	−50.62	768
Umuarama	9	3208	−25.44	−53.28	480
Average	7	2482	-	-	-

**Table 5:** *Diceraeus melacanthus* potential annual generations (PAG) and average municipal accumulated degree-days (AMADD) for 31 municipalities in the São Paulo state, Brazil; temperature data collected from 20 years of weather registry. Londrina, Paraná state, IDR-Paraná, 2025.

São Paulo Municipalities	PAG	AMADD	Latitude	Longitude	Altitude
Ariranha	10	3466	−21.13	−48.84	525
Avaré	8	2669	−23.10	−48.95	775
Barra Bonita	10	3441	−22.37	−48.56	544
Barra do Turvo	5	1803	−24.96	−48.42	667
Barretos	10	3450	−20.56	−48.54	533
Barueri	7	2551	−23.52	−46.87	791
Bauru	9	3171	−22.36	−49.03	666
Campos do Jordão	3	973	−22.75	−45.60	1642
Casa Branca	9	3048	−21.78	−47.08	730
Franca	9	2982	−20.58	−47.38	1026
Ibitinga	9	3241	−21.86	−48.67	492
Iguape	8	2881	−24.72	−47.55	2,66
Itapeva	7	2389	−23.98	−48.89	745
Itapira	9	3045	−22.42	−46.81	633
Ituverava	10	3409	−20.36	−47.77	600
Jales	11	3893	−20.17	−50.59	457
José Bonifácio	11	3595	−21.09	−49.92	405
Lins	11	3624	−21.67	−49.73	450
São Paulo	8	2668	−23.48	−46.62	792.06
Ourinhos	9	3237	−22.95	−49.89	448
Piracicaba	9	3102	−22.70	−47.62	573
Pradópolis	9	3133	−21.34	−48.11	544
Presidente Prudente	11	3733	−22.12	−51.40	435.55
Rancharia	9	3107	−22.37	−50.97	398
São Carlos	8	2785	−21.98	−47.88	863
São Luís do Paraitinga	6	2201	−23.23	−45.42	730
São Miguel Arcanjo	7	2362	−23.85	−48.16	678
Sorocaba	8	2670	−23.35	−47.67	609
Taubaté	8	2750	−23.04	−45.52	571
Valparaíso	11	3716	−21.32	−50.93	374
Votuporanga	11	3751	−20.40	−49.97	465
Average	9	2995			

**Table 6:** ANOVA test results for potential annual generations (PAG) and average municipal accumulated degree-days (AMADD) for Mato Grosso do Sul, Paraná, and São Paulo states, data derived from 25 randomly selected municipalities. Londrina, Paraná state, IDR-Paraná, 2025.

PAG ANOVA					
**Variables**	**Degrees of freedom**	**Sum of Squares**	**Mean Square**	**F**	**F (1%)**
**Treatment**	2	20,958,349.93	10,479,174.97	34.60	4.91
**Residue**	72	21,804,280.47	302,837.23		
**Total**	74	42,762,630.4			
**c.v.**	18.04				
AMADD ANOVA					
**Variables**	**Degrees of freedom**	**Sum of Squares**	**Mean Square**	**F**	**F (1%)**
**Treatment**	2	20,958,349.93	10,479,174.97	34.60	4.91
**Residue**	72	21,804,280.47	302,837.23		
**Total**	74	42,762,630.4			
**c.v.**	18.04				

**Table 7:** Potential annual generations (PAG) and average municipal accumulated degree-days (AMADD) for the Mato Grosso do Sul, Paraná, and São Paulo states, data derived from 25 randomly selected municipalities. Londrina, Paraná state, IDR-Paraná, 2025.

State	PAG *	AMADD *
**Mato Grosso do Sul**	10.99 ± 0.96 a	3750.87 ± 326.32 a
**Paraná**	7.25 ± 1.90 c	2473.32 ± 647.45 c
**São Paulo**	8.58 ± 1.81 b	2929.32 ± 618.74 b
**c.v. (%)**	18.04	18.04

* Means in the column followed by the same letters do not significantly differ according to the Scott–Knott test (α < 0.01).

## Data Availability

The raw data supporting the conclusions of this article will be made available by the authors on request.

## Abbreviations

The following abbreviations are used in this manuscript:

ADD	Accumulated degree-days
AMADD	Average municipality accumulated degree-days
CRS	Coordinate referenced system
CSV	Comma separated values
DD	Degree-days
IDR-PARANÁ	Instituto de Desenvolvimento Rural do Paraná IAPAR-EMATER
INMET	National Institute of Meteorology
PAG	Potential annual generations
